# Studies on Carbon Materials Produced from Salts with Anions Containing Carbon Atoms for Carbon Paste Electrode

**DOI:** 10.3390/ma14102480

**Published:** 2021-05-11

**Authors:** Katarzyna Skrzypczyńska, Andrzej Świątkowski, Ryszard Diduszko, Lidia Dąbek

**Affiliations:** 1 Łukasiewicz Research Network—Industrial Chemistry Institute, 01-793 Warsaw, Poland; katarzyna.skrzypczynska@ichp.pl; 2 Institute of Chemistry, Military University of Technology, 00-908 Warsaw, Poland; andrzej.swiatkowski@wat.edu.pl; 3 Łukasiewicz Research Network—Institute of Microelectronics and Photonics, 01-919 Warsaw, Poland; ryszard.diduszko@itme.edu.pl; 4 Faculty of Environmental, Geomatic and Energy Engineering, Kielce University of Technology, 25-314 Kielce, Poland

**Keywords:** combustion synthesis, salts with carbon in molecule, carbon materials, modified CPEs

## Abstract

In the presented work, the properties of carbon materials obtained in the reaction of sodium bicarbonate (C-SB) and ammonium oxalate (C-AO) with magnesium by combustion synthesis were investigated. For the materials obtained in this way, the influence of the type of precursor on their properties was analyzed, including: Degree of crystallinity, porous structure, surface topography, and electrochemical properties. It has been shown that the products obtained in magnesiothermic process were found to contain largely the turbostratic carbon forming a petal-like graphene material. Both materials were used as modifiers of carbon paste electrodes, which were then used to determine the concentration of chlorophenol solutions by voltammetric method. It was shown that the peak current determined from the registered differential pulse voltammograms was mainly influenced by the volume of mesopores and the adsorption capacity of 4-chlorophenol for both obtained carbons.

## 1. Introduction

The presence of an increasing amount of pollutants in the environment, including micropollutants, entails the development of research in the field of analytical methods. This method should be more sensitive and accurate, and on the other hand, easy to apply, which will allow their use not only in specialized laboratories, but also by various services working for environmental protection. The standardization of the methods used is also important. Among the whole range of analytical methods (classical, including chromatographic, spectrophotometric), electrochemical methods are of significant importance [1], the measurement possibilities of which are related to the availability of specific/selective electrodes. Carbon paste electrodes (CPEs) are of significant importance in this respect due to their simple structure, easy production, low price, and the possibility of multiple uses in electroanalysis. The usefulness of CPEs in electroanalysis can be significantly improved by using their modification consisting in adding a third component to the basic composition of carbon paste (graphite-paraffin/mineral oil) [2]. Carbon modifiers of CPEs (including graphene, carbon nanotubes) have particular advantages, e.g., good contact of their surface with the analyte, high efficiency of analyte accumulation, electrical conductivity.

Research is constantly being carried out on the relationship between their properties and their effectiveness as modifiers. The properties of these materials (carbon modifiers) and the method of their preparation are of significant importance.

One of the methods used is combustion synthesis (self-propagating high-temperature synthesis) with the use of the carbon-containing precursors leads to their destruction and the formation of various carbonaceous materials like carbon nanoparticles [3], carbon encapsulates [4], exfoliated graphite [5]. About ten years ago, we reported the use of chlorine-containing organic compounds as precursors in combustion synthesis initiated by sodium azide [6,7]: The carbon materials thereby obtained possessed unique structural and surface properties, and the nature of the chlorine-containing substrate played an important role. The physicochemical properties of these carbons, crystallinity, porosity, adsorption capacity, electrochemical behavior, surface chemistry, varied according to the kind of organic precursor used. Recently, metallothermic reduction of oxalic acid [8], magnesium oxalate [9] or ammonium oxalate (acetate) [10] was utilized with magnesium for preparing carbon materials. The properties of the obtained carbons were characterized in some detail, generally apart from the porous structure. Their uses have also not been studied. Only in one study [10], the adsorption of 4-chlorophenol was investigated. All products of the magnesiothermic precess [8,9,10] were found to contain largely the turbostratic carbon forming a petal-like graphene material.

Taking all this into account, it could be assumed that these materials could prove to be good modifiers for carbon paste electrodes. It also seemed advisable to use also carbon obtained from inorganic salt, in addition to previously produced only from organic salts.

In this work, carbon material was produced using the described method, based on sodium bicarbonate and ammonium oxalate, and the influence of the type of precursors (anions of the salts used) on the physicochemical properties of the obtained carbons was investigated. At the same time, the usefulness of the so obtained carbon materials as modifiers of carbon paste electrodes (CPE) for the determination of the concentration of organic compounds in an aqueous solution was assessed. 

Among the environmental pollutants, 4-chlorophenol was chosen as an example of an organic pollutant from the group of halogenophenol derivatives with a high degree of toxicity, occurring in the environment in a wide range of concentrations [11,12]. 

## 2. Materials and Methods

In typical a reaction, powdered magnesium (Mg—of particle diameter below 100 µm) and sodium bicarbonate NaHCO_3_ or ammonium oxalate (NH_4_)_2_C_2_O_4_ were triturated dry in ceramic mortar, to obtain homogenic mass. Pressed sample of mass about 10 g was placed in steel crucible, and the combustion process was operated in a steel reactor of volume 275 cm^3^, filled with argon under pressure 10 bar. Combustion was carried out thermoelectrically with resistance wire held to the surface of substrate mixture. Results of calorimetric measurements gave values of reaction heats as follows (1,2):

3Mg + NaHCO_3_ → 3MgO + C + NaH         Q = 4692 J/g (1)

5Mg + (NH_4_)_2_C_2_O_4_*H_2_O → 5MgO + 2C + 2NH_3_ + 2H_2_  Q = 4344 J/g (2)

Raw reaction products were cleaned by hours of leaching with concentrated hydrochlorid acid, and next boiling in water. Obtained carbon materials were further referred to as C-SB (sodium bicarbonate) and C-AO (ammonium oxalate).

### 2.1. Diffraction Analysis

X-ray diffraction (XRD) pattern was measured (D500 Diffractometer, Siemens, Karlsruhe, Germany) using Cu Kα radiation, in the 2*Θ* range 10–60° for raw reaction products and for cleaned reaction products with a 0.05° step.

### 2.2. Porosity and Texture

The porosity of the cleaned combustion products was characterized by low-temperature nitrogen adsorption, the relevant isotherms of all samples were measured at 77.4 K on the ASAP 2010 volumetric adsorption analyzer (Micromeritics, Norcross, GA, USA). Before each adsorption measurement, the sample was outgassed under vacuum at 200 °C. Scanning electron micrograph study was performed with using DSM 942 (Carl Zeiss, Jena, Germany) scanning electron microscope (SEM) for EHT = 2.00 kV. 

### 2.3. Voltammetric Investigations

The electrochemical experiments were carried out using an Autolab potentiostat/galvanostat (model PGSTAT 20, Eco Chemie B.V., Utrecht, The Netherlands) connected to a desktop computer and controlled by a GPES 4.9 software(Eco-Chemie, Utrecht, Netherland. All experiments were carried out in a conventional three-electrode system. The electrode system contained as working electrode carbon paste electrode, a platinum wire as a counter electrode, and a saturated calomel electrode as a reference electrode.

### 2.4. Preparation of Carbon Paste Electrodes

The bare carbon paste electrode was prepared by mixing 65% of graphite powder (diameter < 20 μm) and 35% of high purity paraffin oil (both components from Sigma-Aldrich, St. Louis, MO, USA) in an agate mortar by hand mixing for about 20 min to get homogenous carbon paste. The paste was packed into the cavity of Teflon electrode body and smoothened on weighing paper.

The modified carbon paste electrodes [2] were prepared by mixing graphite powder and modifier (5 or 10%) with paraffin oil. Homogenization is then achieved by careful mixing using agate pestle and mortar and afterwards rubbed by intensive pressing with the pestle. The mixture was kept at room temperature for two days. The ready-prepared paste was then packed into the hole of the electrode body and the carbon paste was smoothed onto a paper until it had a shiny appearance.

## 3. Results and Discussion

### 3.1. Structure and Porosity of Obtained Carbon Materials

**Figure 1:**
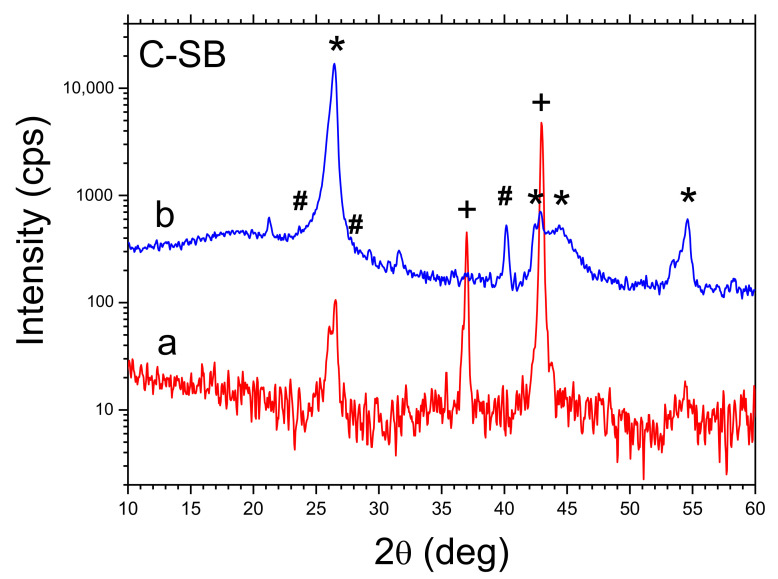
XRD patterns of C-SB (sodium bicarbonate), a—raw reaction product, b—cleaned. Symbols: * graphite, + MgO, # Mg_2_Si.

**Figure 2:**
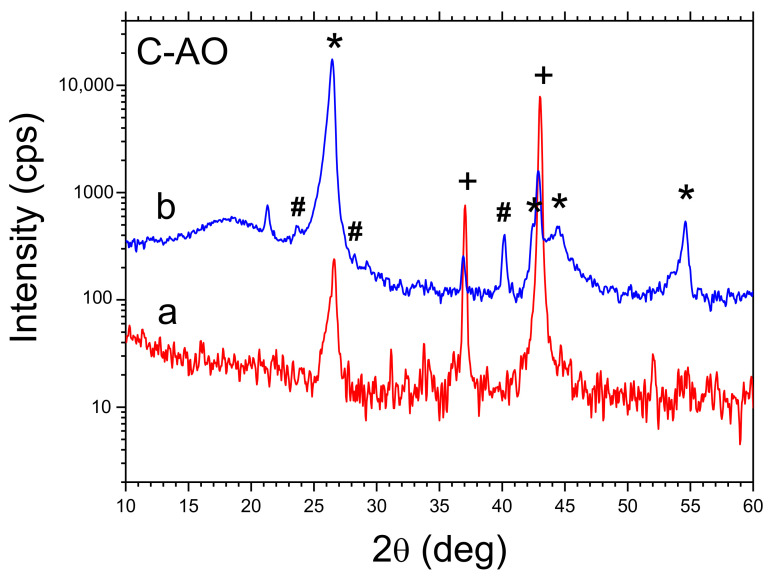
XRD patterns of C-AO (ammonium oxalate), a—raw reaction product, b—cleaned. Symbols: * graphite, + MgO, # Mg_2_Si.

XRD spectra recorded for as obtained raw carbon materials C-SB and C-AO as well as cleaned (HCl, H_2_O) ones presented in Figure 1 and Figure 2 revealed effects of byproducts removing.

It has been shown that the cleaning procedure used effectively removes the MgO compound from the structure (in the case of CA-O a trace amount remains, perhaps due to the occlusion phenomenon), while in both cases, a trace amount of Mg_2_Si appears in the structure. The calculated parameters characterized the structure of both carbon materials (interlayer spacing of crystalline structure d_002_ and number of layers NC) are collected in Table 1. For comparison, in Table 1, both parameter values for graphite G-SA used in CPEs are shown. These data show that the structure of both obtained carbon materials is intermediate between typical for graphite and carbon black [13,14,15,16]. The values of the interlayer spacing d_002_ of the crystal structure and the number of layers NC presented in Table 1 show relatively small differences of these parameters for both carbons (C-AO and C-SB). Significant differences between the parameters characterizing the porous structure of both obtained carbons concern mainly the specific surface area and the volume of the mesopores, for C-AO their values are respectively 1.28 and 2.31 times greater than for C–BS.

**Figure 3:**
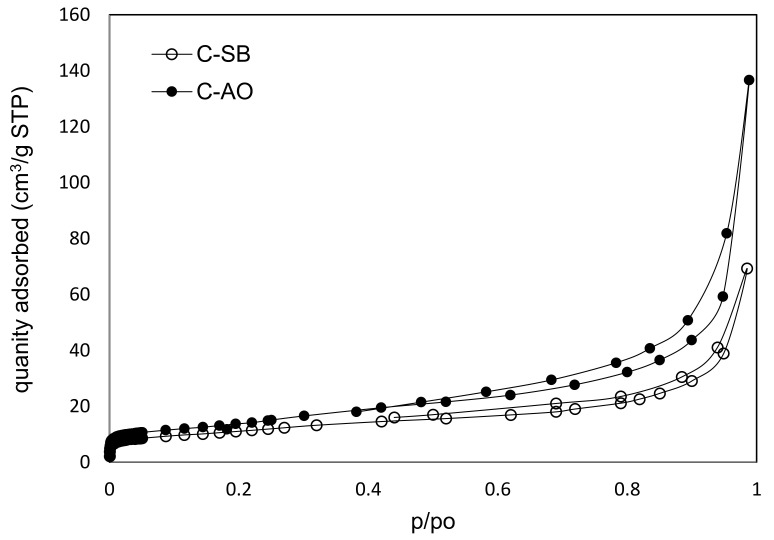
Nitrogen adsorption-desorption isotherms on cleaned carbon materials at 77 K.

**Figure 4:**
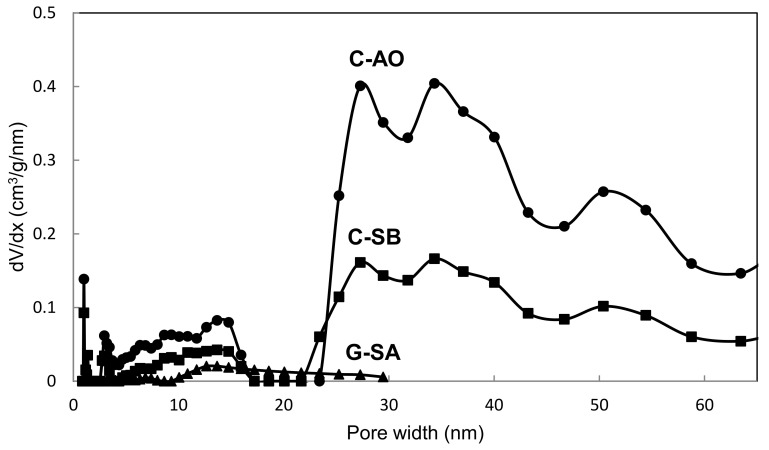
Pore size distribution of obtained cleaned carbon materials as well as graphite used in CPEs.

The parameters of the porous structure of the obtained carbons C-SB and C-AO, i.e., the specific surface areas (S_BET_) and micropore volumes (V_mi_) as well as mesopore volumes (V_me_) calculated from determined low-temperature nitrogen adsorption isotherms (Figure 3 and for G-SA in [17]) are presented in Table 1. The values of BET surface areas, as well as micro- and mesopores volumes, are less than for typical carbon blacks or graphitized carbon blacks [15] but higher than for graphite. Pore size distribution for both carbon materials, as well as for graphite used in carbon paste electrodes, are shown in Figure 4. The pore size distribution for carbons C-AO and C-SB shows similarity for both carbons and reflects the differences in their mesopore volumes. The major part of the mesopore volume ranges in size from about 23 to 50 nm and further enters the macropore region. The second narrower range of mesopore sizes is 2–17 nm, which, however, has a much smaller pore volume.

**Figure 5:**
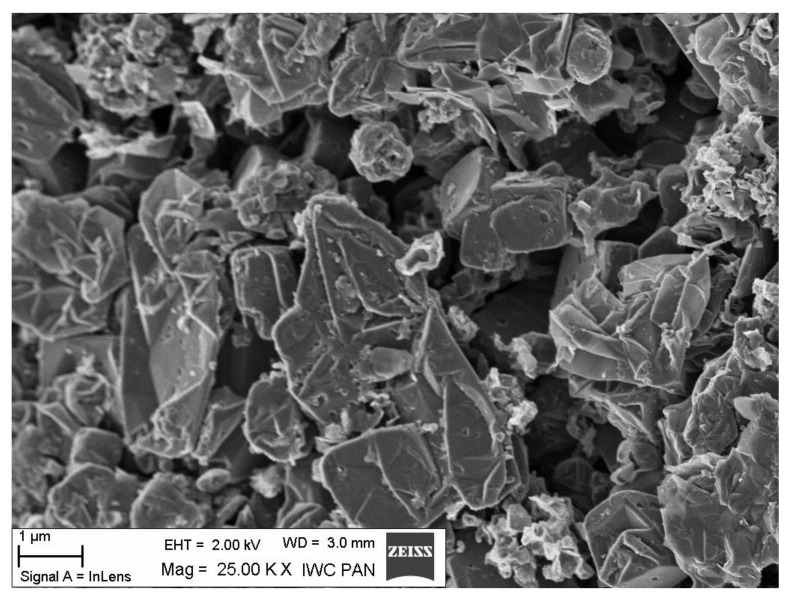
SEM image of carbon material C-SB obtained from NaHCO_3_.

**Figure 6:**
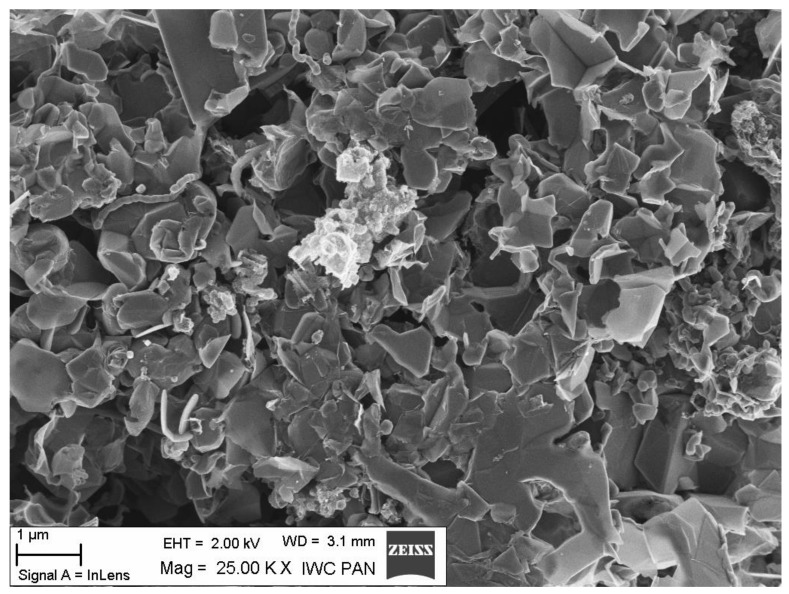
SEM image of carbon material C-AO obtained from (NH_4_)_2_C_2_O_4_.

Surface texture shown in SEM micrographs is given in Figure 5 and Figure 6. The SEM images of both carbon materials are similar and represent a petal-like grapheme material. 

### 3.2. Influence of the CPE Modifier on the Voltammetric Measurements Results

**Figure 7:**
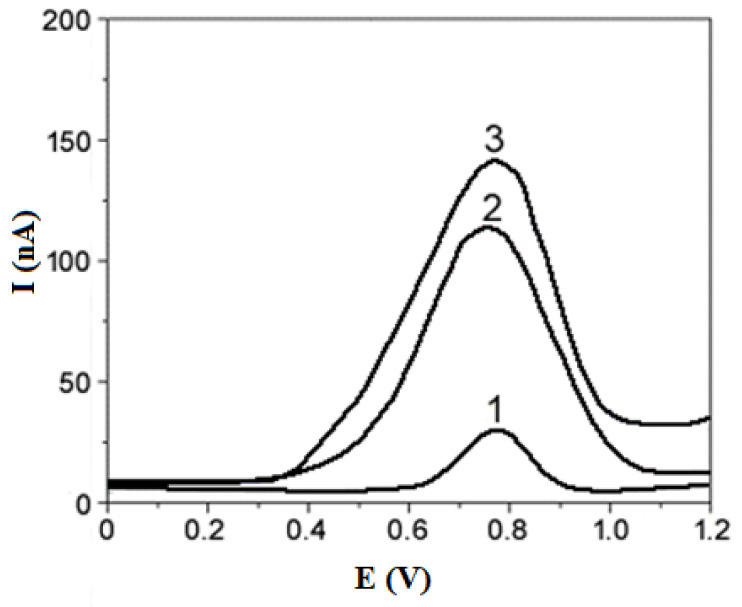
DPV curves registered for 0.5 mmol/dm^3^ 4-CP solutions in 0.1 mol/dm^3^ Na_2_SO_4_ using carbon paste electrodes containing 10 wt.%. of tested materials-modifiers: 1—bare CPE, 2—C-SB, 3—C-AO.

Differential pulse voltammograms (DPV) for the modified CPEs as an example with 0.5 mmol/L 4-CP solutions are shown in Figure 7.

**Figure 8:**
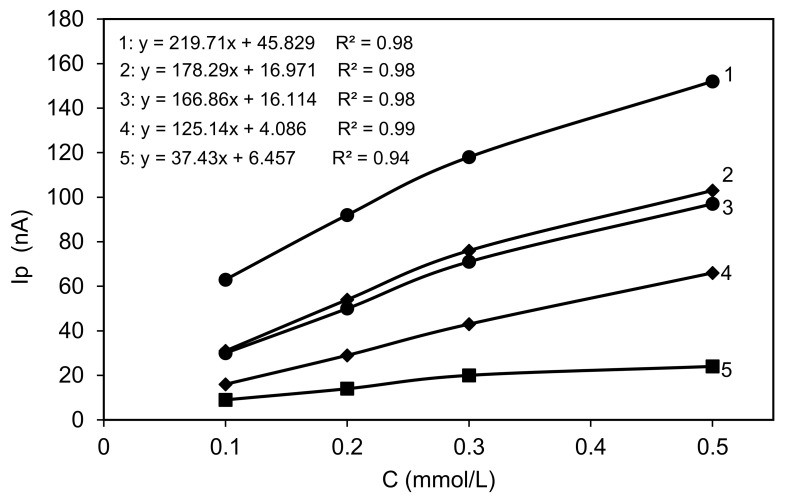
The dependence of peak current in DPV curves on 4-CP concentration for: 1—C-AO 10 wt.%, 2—C-SB 10 wt.%, 3—C-AO 5 wt.%, 4—C-SB, 5 wt.%, 5—bare CPE.

From all the DPV curves (CPEs without as well as with 5% and 10 wt.%. modifiers content) the peak currents I_p_ and the peak potentials E were determined. All the obtained values are presented in Table 2. The peak potentials reveal slightly different values from 0.78 to 0.79 V for all the used 4-CP concentrations and both modifier’s contents or the unmodified CPE. The recorded DPV curves show the dependence of the peak current on the 4-CP solution concentration (Figure 8).

The calibration relationships (peak current versus 4-chlorophenol concentration) were fitted for all CPEs by linear least squares regression analysis. The equations obtained by us, as well as the regression coefficients, are presented in Figure 8. A linear relationship is observed for all CPEs (R^2^ > 0.98). The I_p_ exhibit increasing values with an increasing 4-CP concentration for each of the used content of CPE modifier. The peak currents in the case of each modified CPE are about 1.5–2 times higher when the 4-CP concentration is increased two times. The results collected in Table 2 also show the other dependence. The increase of the CPE modifier content gives an increase in the peak current. When the modifier content was enhanced (from 5% to 10 wt.%) one can observe a peak current increase of about 1.7 times. Significant differences in peak current also give the kind of used modifier, obtained carbon material. For each of both contents of modifier and each used 4-CP concentration the C-AO gives higher I_p_ values than C-SB by about 1.5–1.7 times. The specific surface area of C-AO is about 1.3 times higher than of C-SB. The V_mi_ of both carbons are almost equal but V_me_ is about two times higher for C-AO. It means that the width of pores is important (for both carbon materials in the range of about 25–70 nm).

Similar dependence I_p_ on S_BET_ was previously observed in the case of activated carbon and carbon black materials used as CPE modifiers [16,18]. Increasing the surface area enhanced peak currents for CPE in 4-chlorophenol solutions. For another type of electrode, the powdered carbon electrodes in the case of cyclic voltammetric measurements in 4-CP solutions, such kind of dependence was also observed [15].

Another interesting observation concerns the value of the current I_p_ measured for both carbon materials produced in this work. In our earlier work [16] for the electrode modified with the addition of Vulcan XC72 carbon black (6%) and for the 0.5 M solution of 4-chlorophenol, the peak current value was 79 nA. However, in this work, for 5% of additives of C-SB and C-AO carbon materials as modifiers, the current values were 68 and 97 nA, respectively. So they were similar to those for the XC72 carbon black used as a modifier. This was despite the fact that this carbon black has a specific surface area approximately 4.6 and 5.9 times greater than that of carbons C-AO and C-SB, respectively. This may be due to the difference in the morphology of the carbon black and our carbon materials.

Vulcan XC72 carbon black has an intermediate structure between amorphous and graphitic, called turbostratic structure. The layers of carbon blacks are parallel to each other but not arranged in order, usually forming concentric inner layers. Carbon black is composed of carbon primary spherical particles of diameters about 30 nm fused together by covalent bonds, thus forming aggregates. Several aggregates can interact to give place to a secondary structure known as agglomerate [19].

Comparing the properties of the above-mentioned carbon black [16] and the carbons obtained in this work, it can be seen that the S_BET_ value is not a proper parameter influencing the measured I_p_ of CPE. The analysis of other parameters showed that the V_me_ of the carbon black is only 1.38 times greater than the C-AO. Another property of the compared carbon materials, i.e., the adsorption capacity of 4-chlorophenol, also shows a great similarity, e.g., for carbon black and carbon C-AO it is close to 0.5 mmol/g, which was determined, respectively, in the works [10,16]. In recent works on modified carbon paste electrodes [20], carbon nanotubes and reduced graphene oxide have been presented as frequently used modifiers. The products obtained in magnesiothermic process were found to contain largely the turbostratic carbon forming a petal-like graphene material. These materials show promise as modifiers for carbon paste electrodes due to the good contact of their surface with the analyte.

## 4. Conclusions

The aim of the work was to demonstrate that the proposed type of carbon materials can be successfully used as a modifier of carbon paste electrodes for the determination of chlorophenols concentration by electrochemical method.

Compared to other works on obtaining such specific carbon materials by combustion synthesis, the carbons analyzed in this work were obtained using organic and also inorganic salts containing carbon in their anions, which gives them specific features. It has been shown and confirmed that the products obtained in magnesiothermic process were found to contain largely the turbostratic carbon forming a petal-like graphene material. They were characterized by a relatively small specific surface area and volume of micropores, but a relatively large volume of mesopores. This was shown much better for carbon obtained from ammonium oxalate. The peak current, indicating the effectiveness of the carbon paste electrode modifier, recorded by the DPV method for this carbon as the CPE modifier was comparable to that determined for the carbon black with a specific surface area of 4.5 times greater. This indicates that the surface area and volume of the micropores cannot be the only parameters taken as parameters determining the value of the peak current. The usefulness of carbons produced by the magnesiothermic method as effective CPE modifiers has been used to determine the concentration of 4-chlorophenol.

This encourages the use of these electrodes for the determination of other pollutants as well, which may also contribute to the wider use of electrochemical methods in the analysis of environmental samples.

## Figures and Tables

**Table 1:** Characteristics of structure and porosity of obtained carbon materials.

Carbon Material	d_002_ (nm)	NC	S_BET_ (m^2^/g)	C	V_mi_ (cm^3^/g)	V_me_ (cm^3^/g)
G-SA	0.3357	>200	4.5	160	0.0008	0.007
C-SB	0.3362	62	39	137	0.005	0.062
C-AO	0.3361	66	50	110	0.005	0.143

**Table 2:** Peak currents and potentials determined from DPV curves for carbon paste electrodes modified by adding various quantity of obtained carbon materials.

Modifier Materials	Modifier Content (%)	Concentrations of 4-Chlorophenol Solutions (mmol/L)				
		Peak Current (nA)				Peak Potential (V)
		0.50	0.30	0.20	0.10	
bare CPE	-	24	20	14	9	0.79 ± 0.02
C-SB	5	68	41	29	18	0.79 ± 0.02
	10	103	76	52	31	0.79 ± 0.02
C-AO	5	97	71	50	32	0.78 ± 0.02
	10	152	121	87	57	0.78 ± 0.02

## Data Availability

Data sharing is not applicable to this article.
